# Identification of brain cell types underlying genetic association with word reading and correlated traits

**DOI:** 10.1038/s41380-023-01970-y

**Published:** 2023-02-07

**Authors:** Kaitlyn M. Price, Karen G. Wigg, Anukrati Nigam, Yu Feng, Kirsten Blokland, Margaret Wilkinson, Elizabeth N. Kerr, Sharon L. Guger, Maureen W. Lovett, Lisa J. Strug, Shreejoy J. Tripathy, Cathy L. Barr

**Affiliations:** 1 Division of Experimental and Translational Neuroscience, Krembil Research Institute, University Health Network, Toronto, ON Canada; 2 Program in Neuroscience and Mental Health, Hospital for Sick Children, Toronto, ON Canada; 3 Department of Physiology, University of Toronto, Toronto, ON Canada; 4 Krembil Centre for Neuroinformatics, Centre for Addiction and Mental Health, Toronto, ON Canada; 5 Institute of Medical Science, University of Toronto, Toronto, ON Canada; 6 Department of Psychology, Hospital for Sick Children, Toronto, ON Canada; 7 Department of Pediatrics, University of Toronto, Toronto, ON Canada; 8 Genetics and Genome Biology, Hospital for Sick Children, Toronto, ON Canada; 9 Departments of Statistical Sciences and Computer Science, Faculty of Arts and Science and Division of Biostatistics, Dalla Lana School of Public Health, University of Toronto, Toronto, ON Canada; 10 Department of Psychiatry, University of Toronto, Toronto, ON Canada

**Keywords:** ADHD, Genetics

## Abstract

Neuroimaging studies implicate multiple cortical regions in reading ability/disability. However, the neural cell types integral to the reading process are unknown. To contribute to this gap in knowledge, we integrated genetic results from genome-wide association studies for word reading (n = 5054) with gene expression datasets from adult/fetal human brain. Linkage disequilibrium score regression (LDSC) suggested that variants associated with word reading were enriched in genes expressed in adult excitatory neurons, specifically layer 5 and 6 FEZF2 expressing neurons and intratelencephalic (IT) neurons, which express the marker genes LINC00507, THEMIS, or RORB. Inhibitory neurons (VIP, SST, and PVALB) were also found. This finding was interesting as neurometabolite studies previously implicated excitatory-inhibitory imbalances in the etiology of reading disabilities (RD). We also tested traits that shared genetic etiology with word reading (previously determined by polygenic risk scores): attention-deficit/hyperactivity disorder (ADHD), educational attainment, and cognitive ability. For ADHD, we identified enrichment in L4 IT adult excitatory neurons. For educational attainment and cognitive ability, we confirmed previous studies identifying multiple subclasses of adult cortical excitatory and inhibitory neurons, as well as astrocytes and oligodendrocytes. For educational attainment and cognitive ability, we also identified enrichment in multiple fetal cortical excitatory and inhibitory neurons, intermediate progenitor cells, and radial glial cells. In summary, this study supports a role of excitatory and inhibitory neurons in reading and excitatory neurons in ADHD and contributes new information on fetal cell types enriched in educational attainment and cognitive ability, thereby improving our understanding of the neurobiological basis of reading/correlated traits.

## Introduction

Reading Disability (RD) is a common neurocognitive disorder, resulting in difficulties predominantly with word reading [1]. It overlaps both clinically and genetically with other neurodevelopmental disorders [2]. Together, these difficulties impact academic achievement and subsequent employment opportunities, resulting in life-long sequelae.

RD is described as a complex polygenic trait, influenced by many genetic factors [3, 4]. Genetic regions were initially elucidated by family-based linkage and fine-mapping association studies [5]. This research gave way to more powerful genome-wide association studies (GWAS). The majority of GWAS results, so far, do not reach the threshold for genome-wide significance nor explain the heritable variance, largely due to sample size. However, as Consortia are established and samples grow, significant results are beginning to reach statistical significance. For example, *RPL7P34* (p~10^−8^) [6], *MIR924HG* (p~10^−9^) [7], and *DOCK7* (p~10^−8^) [8, 9] were significantly associated with reading or reading component skills in large samples. Further, 42 loci for self-reported dyslexia were recently identified in the 23andme cohort [3].

The mechanisms of action by which preliminary (p~10^−7^)/significantly associated genetic variants act are largely unknown. A challenge of the post-GWAS era is linking single nucleotide polymorphisms (SNPs) to risk genes and then to a molecular mechanism that influences cellular and brain function [10]. Typically, associated variants are located in the non-coding region of the genome, introns or intergenic, and do not directly change the protein code [11, 12]. Associated variants or their linkage disequilibrium (LD) neighbors are often found in regulatory elements (e.g., promoters, enhancers) marked by DNAseI hypersensitivity sites, transcription factor binding sites, or histone methylation/acetylation sites [10, 11]. This positioning suggests a role in controlling gene expression [11–13]. However, the genes that the risk variants influence may be located millions of base pairs away [14], further complicating the interpretation of GWAS findings. Another factor complicating the annotation of risk variants is that regulatory elements operate in a tissue/cell type specific manner; this presents a major challenge, as we do not know the relevant nor specific cell types for reading ability/disability.

Reading requires multiple cognitive processes and multisensory integration of visual symbols with their corresponding speech sounds [15–18]. Consequently, multiple brain regions, mostly cortical, have been implicated in reading as indicted by neuroimaging studies [19–24]. These regions include the anterior system, Broca’s area located in the inferior frontal gyrus [22, 23], and the posterior systems, dorsal parietotemporal and ventral occipitotemporal systems [20]. As for the cell types within these regions, generally speaking, neurons are thought to be involved. The leading theory for RD etiology states that risk variants contribute to subtle disruptions in neuronal migration, which lead to altered connectivity of language-related brain regions [25, 26]. Known as the disrupted neuronal migration (DNM) hypothesis, this theory was developed through post-mortem studies that found left-hemisphere polymicrogyria in the planum temporale of individuals with RD, indicative of DNM (reviewed by [25]), as well as heterotopias, dysplasias, and dyslamination [27–29]. Genetic studies have also provided some support for this hypothesis (reviewed by [25]).

Cortical neurons, either glutamatergic (excitatory) or GABAergic (inhibitory), are likely candidates and glial cells may also be involved [30, 31]. Excitatory-inhibitory imbalances have been implicated in RD and genetically correlated traits. Using magnetic resonance spectroscopy (MRS), RD studies measuring neurometabolites have identified increased cortical glutamate, with higher concentrations correlated with lower reading skills [32–34]. High levels of glutamate were also found for autism spectrum disorder (ASD) [35–38] and ADHD [39–41]. These data prompted the neural noise hypothesis [34, 42], which posits that increased glutamate leads to neural hyperexcitability -- in this case, in networks supporting reading [34].

The neural noise hypothesis and DNM are intrinsically linked. The brain needs correct neuronal migration for functional cortical circuit formation and excitatory-inhibitory synaptic connections [34]. Similarly, excitatory projections provide cues to properly position inhibitory interneurons. Therefore, just as neuronal migration may disrupt excitatory-inhibitory balances, excitatory-inhibitory balances may disrupt migration [43]. Recently, researchers showed that hyperexcitability led to changes in gene expression, which decreased mature neuron markers and increased immature markers, demonstrating the connectedness of excitability and migration. This was shown in the context of schizophrenia, Alzheimer’s, and amyotrophic lateral sclerosis [44].

Determining relevant cell types is crucial for understanding the genetic and molecular mechanisms of reading and reading failure, but are largely unknown. In this study, we used linkage disequilibrium score regression (LDSC) [45, 46] to test whether GWAS heritability is enriched for particular adult and fetal brain cell types. This method leverages significant/non-significant GWAS findings and gene expression data to better interpret GWAS results and prioritize cell types for downstream functional investigation. We used a word reading GWAS as well as large GWAS for ADHD, educational attainment, and cognitive ability to improve power. In previous studies, we and others, identified shared genetic etiology between word reading and ADHD, educational attainment, and cognitive ability using polygenic risk scores [7, 47]. Genetic correlation between these traits was also identified by Eising et al. (2022) [4] (reading/spelling measures vs. educational attainment or full-scale IQ r^2^ ~/> 0.5) and Doust et al. (2022) [3] (self-reported dyslexia vs. educational attainment or measures of IQ r^2^ ~ -0.2). Because the traits share genetic overlap, understanding cell type enrichment in ADHD, educational attainment, and cognitive ability will complement our analysis of word reading.

Previous studies examined ADHD, educational attainment, and cognitive ability using different adult and fetal RNA sequencing data [48–50]. We add to these findings by using different datasets, confirming previous findings, and identifying novel cell types for these traits.

## Materials and methods

**Fig. 1:**
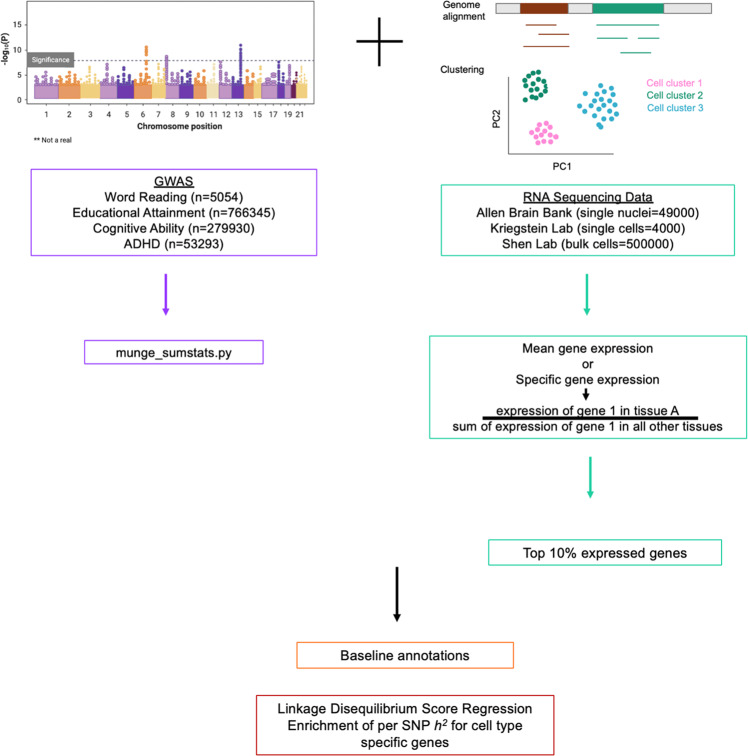
Input for LDSC. For LDSC analysis, GWAS summary statistics for word reading or related traits, educational attainment, cognitive ability, and ADHD, were used. The command munge_sumstats.py converted summary statistic files from.txt to.sumstats.gz files. Single cell and bulk RNA sequencing gene expression data was used to make the annotation files. Mean expression or specific expression was calculated. Only the top 10% most expressed or specific genes were used for LDSC. Baseline annotations were included as controls.

We used the LDSC method ‘Partitioning Heritability’ (https://github.com/bulik/ldsc/wiki/Partitioned-Heritability), which incorporates GWAS summary statistics, gene expression data, and baseline annotations as genomic controls (Fig. 1 [BioRender - adapted from Bailey Harrington and [13]]) to test whether the heritability of a particular cell type, defined by its highest mean expressed genes, is significantly contributing to overall SNP heritability of a trait [45, 46]. Results from a previous study indicate that for the most part, results from LDSC, DEPICT, and MAGMA (top 10% mode) identify similar cell types [50]. Based on those findings, we used a single cell type enrichment tool.

### GWAS datasets

A meta-analysis of two GWAS for word reading was used in this study, which has previously been described [47]. Briefly, the samples in the meta-analysis consisted of a family-based sample from Toronto (*n* = 624) and a population-based sample from Philadelphia (Philadelphia Neurodevelopmental Cohort (PNC), *n* = 4430). The Toronto sample recruited child with reading difficulties and their siblings from Ontario, Canada [47, 51–53]. Children were assessed for multiple measures of reading and language at the Hospital for Sick Children. All participants gave informed consent or assent. Procedural approval was given by the Hospital for Sick Children and University Health Network Research Ethics Boards.

The PNC was recruited through a NIMH-funded Grant Opportunity and sought to characterize neurobehavioral traits in genotyped children from the community [54]. All children were recruited from the Children’s Hospital of Philadelphia (CHOP) or CHOP-affiliated clinics [55, 56] with measures of word reading. The PNC genotype and phenotype information were downloaded after approval from dbGAP (Neurodevelopmental Genomics: Trajectories of Complex Phenotypes Cohort).

For both samples, unobserved genotypes were imputed using the Michigan Imputation Server with the Haplotype Reference Consortium (version r1.1) [57]. Quality control was performed by removing SNPs with a low imputation quality score (*r*^2^ < 0.30), out of Hardy-Weinberg equilibrium (p < 0.0001), and with a low minor allele frequency (MAF) (< 5%). Only individuals self-identified as European ancestry were included in the analysis and mapped using principal component analysis (PCA). The GWAS were performed using linear mixed models to account for family relationships. Both samples had versions of the same reading measure, the Wide Range Achievement Test (WRAT) 3 or 4. For the Toronto sample the WRAT3 was the outcome variable and for the PNC, the WRAT4. Principal components for population structure and genotypes were included as fixed effects, and family relationship was included as a random effect. After completing the two linear mixed models, a meta-analysis was performed on the Toronto and PNC samples using METAL [58].

The reading-related summary statistics for ADHD [59], educational attainment [60], and cognitive ability [61] were downloaded from the Psychiatric Genomics Consortium website https://www.med.unc.edu/pgc/download-results/ or https://ctg.cncr.nl/software/summary_statistics or https://www.thessgac.org/. These summary statistics included individuals with European ancestry determined by PCA.

### GWAS dataset processing for LDSC

For each set of summary statistics, SNPs were removed with low MAF (<1%) and a low imputation quality score <0.7 (exception: educational attainment and word reading, score <0.3). SNPs within the major histocompatibility region (chr6: 26–33 Mb) were also removed due to high LD. The LDSC command ‘munge_sumstats.py’ was used to munge summary statistics into ‘.sumstat.gz’ to prepare for further analysis.

### Heritability of GWAS datasets

To partition heritability in LDSC, adequate GWAS level SNP-based heritability is required. It is estimated to be a z-score (*h*^*2*^/se) of greater than 7 [46, 62], although leniency is accepted. To measure *h*^2^ and standard error to compute z-scores, the LDSC ‘ldsc.py’ command was used with –h2/–rg.

### Gene expression datasets

Gene expression datasets from the Allen Brain Bank (ABB) [63], the Kriegstein lab [64], and the Shen lab [65] were used, consisting of single nucleus RNA sequencing (snRNA-seq), single cell RNA seq (scRNA-seq), and bulk RNA seq from flow sorted cells, respectively. These datasets were selected to survey several types of neural cells in fetal and adult brain and across key stages of cortical development. In order to use gene expression datasets as input for LDSC, the datasets are made into an annotation file. The following sections will cover the details of this process.

### Allen Brain Bank dataset

The ABB smart seq snRNA-seq expression data was downloaded from the Allen Brain Bank portal (tome file) (https://transcriptomic-viewer-downloads.s3-us-west-2.amazonaws.com/human/transcriptome.zip). The data set includes ~49,000 dissociated and sorted nuclei using the neuronal marker NeuN (sorts neuronal versus non-neuronal cells). Tissue was collected from adult control post-mortem brains or removed during neurosurgeries (non-psychiatric conditions) [63], from individuals aged 16 to 68 years old. Tissue was collected from the middle temporal gyrus and the anterior cingulate, primary visual, primary motor, primary somatosensory, and primary auditory cortices. Primary analyses were conducted on the four major cell classes (excitatory and inhibitory neurons, oligodendrocytes, and astrocytes, plus non-neural) followed by analyses of 19 subclasses (Supplementary Table 1).

The ABB processing and quality control of all three datasets was conducted in R/R studio v 4.1.0. The Allen Institute R package *scrattch.io* was used to process the ABB dataset. Pseudo-bulking was conducted using cluster label information and removing donor-specific/outlier labels. Note that cluster label is generally defined by gene markers and laminar distribution, although the labels themselves are heterogeneous and position alone could not predict neuron type [63]. Cell types with too few nuclei (<100) were removed including microglia and vascular and leptomeningeal cells. Non-neural cells (endothelial cells, pericytes) were included in the analysis. It was not expected that they would be enriched in reading/related traits.

### Kriegstein dataset

The Kriegstein dataset [64] scRNA-seq data is available on dbGAP (Study of Human Developmental Neurogenesis (phs000989.v4.p1)) and can be used with permission. Raw count data were kindly sent to us from the Kriegstein lab. The data set included ~4000 cells from the primary cortical (visual (V1) and prefrontal cortex (PFC)) and medial ganglionic eminence (MGE) across stages of peak neurogenesis, 5 to 37 post-conception weeks. Tissue was collected from 48 fetal samples. For the analyses, 12 cell classes were used, identified from the supplementary Fig 2a from the Nowakowski et al. (2017) manuscript that contained at least 100 cells (Supplementary Table 2). Due to low cell count, we excluded striatal neurons, early radial glial, early born excitatory neurons, intermediate progenitor cells (radial glial-like), and dividing intermediate progenitor cells (radial glial-like). Cells with no label in the meta-data or ‘unidentified’ were also removed.

### Shen dataset

The Shen dataset [65] was downloaded from Neuroscience Multi-Omic Archive (NeMO Archive) after approval (https://assets.nemoarchive.org/dat-uioqy8b). Cells were isolated from mid-gestational, 15 to 22 gestational weeks, from 17 samples of the human cortex. The Shen lab cells were FAC sorted excitatory neurons, interneurons, radial glia, and intermediate progenitor cells (5×10^5^ to 1.5×10^6^ cells). Bulk RNA-seq were performed on these cells after depletion of ribosomal transcripts. We used the embargo raw expected counts (RSEM).

### Quality control

For the ABB and Kriegstein datasets, cell types with fewer than 1000 genes per nuclei/cell were removed, as were those with greater than 10% expression of mitochondrial or ribosomal genes. For both datasets, the R package *Seurat* was used to construct violin plots and check for outliers.

The Shen data is bulk RNA seq on flow-sorted cells and underwent different quality control. Lowly expressed genes were removed using a cut-off < 10 counts in <3 cells. Like the other datasets, bulk seq was also checked for cells with an over-representation of mitochondrial or ribosomal genes.

### Annotation file

Following the recommendation of LDSC, we used the most highly expressed genes to create the annotation file for each cell type [45, 46]. We computed mean gene expression, primary analysis, and then specificity, secondary analysis, for each gene in each cell type. Specificity is the expression in one cell type divided by the sum of expression in all cell types [45, 46, 48]. We chose to use the mean as our primary analysis and not specificity (supplementary materials) because 30-50% of the 25,000 known protein-coding genes are expressed in brain across all regions [66–69]. The mean or specific gene expression files were separated into deciles using the *cut2* function in Hmisc package. Only the top decile was used.

An R package called *EWCE* (https://github.com/NathanSkene/EWCE/) includes functions to drop lowly expressed genes or genes that do not differ between cell types, and to calculate mean (Tables 1–3) and specificity values (Supplementary Tables 3-5) per gene [48]. This package requires raw single cell count data as input (SCE matrix). For the bulk RNA-seq data, mean and specificity were calculated in R without *EWCE* and no additional genes were dropped after our original quality control (Table 4; Supplementary Table 6).

**Table 1:** Cell type enrichment in ABB adult dataset major class.

GWAS	Major Class	Coefficient	Std Error	*P*-value	FDR
Word Reading	Excitatory	6.28E−08	2.97E−08	1.73E−02	**3.46E−02**
	Inhibitory	6.75E−08	3.01E−08	1.24E−02	**2.76E−02**
	Oligo	2.44E−08	3.25E−08	2.26E−01	3.23E−01
	Astrocyte	4.62E−08	2.95E−08	5.86E−02	1.07E−01
	Non-neural	1.88E−08	3.55E−08	2.98E−01	3.86E−01
ADHD	Excitatory	4.47E−09	4.41E−09	1.55E−01	2.38E−01
	Inhibitory	1.38E−09	4.38E−09	3.76E−01	4.42E−01
	Oligo	−1.70E−09	4.32E−09	6.53E−01	7.23E−01
	Astrocyte	−2.20E−09	4.50E−09	6.87E−01	7.23E−01
	Non-neural	−4.87E−09	4.36E−09	8.68E−01	8.68E−01
Education	Excitatory	6.37E−09	9.21E−10	2.30E−12	**4.60E−11**
	Inhibitory	5.99E−09	9.14E−10	2.87E−11	**2.87E−10**
	Oligo	3.64E−09	1.00E−09	1.45E−04	**4.83E−04**
	Astrocyte	3.01E−09	1.07E−09	2.50E−03	**6.25E−03**
	Non-neural	1.40E−09	1.01E−09	8.29E−02	1.38E−01
Cognitive	Excitatory	1.12E−08	2.07E−09	3.00E−08	**1.50E−07**
	Inhibitory	1.24E−08	2.09E−09	1.74E−09	**1.16E−08**
	Oligo	8.87E−09	2.24E−09	3.73E−05	**1.49E−04**
	Astrocyte	6.46E−09	2.25E−09	2.03E−03	**5.80E−03**
	Non-neural	1.13E−09	2.28E−09	3.09E−01	3.86E−01

Bold indicates significant at FDR .05.

**Table 2:** Cell type enrichment in ABB adult dataset subclass.

GWAS	Major Class	Subclass	Coefficient	Std Error	*P*-value	FDR
Word Reading	Excitatory	L6b FEZF2	8.16E−08	2.94E−08	2.78E−03	**5.56E−03**
	Excitatory	L5/6 NP FEZF2	9.06E−08	3.17E−08	2.14E−03	**4.59E−03**
	Excitatory	IT LINC00507 THEMIS RORB	7.68E−08	2.93E−08	4.42E−03	**8.49E−03**
	Excitatory	L6 CT FEZF2	7.80E−08	2.99E−08	4.53E−03	**8.49E−03**
	Excitatory	L5/6 IT Car3 THEMIS	6.82E−08	2.88E−08	8.84E−03	**1.61E−02**
	Excitatory	L5 ET FEZF2	5.31E−08	3.03E−08	4.01E−02	6.33E−02
	Excitatory	L4 IT RORB	5.03E−08	3.00E−08	4.65E−02	7.15E−02
	Inhibitory	PAX6	4.16E−08	3.14E−08	9.29E−02	1.33E−01
	Inhibitory	LAMP5	3.86E−08	3.13E−08	1.08E−01	1.47E−01
	Inhibitory	VIP	5.81E−08	2.96E−08	2.49E−02	**4.27E−02**
	Inhibitory	SST	5.58E−08	2.92E−08	2.80E−02	**4.54E−02**
	Inhibitory	PVALB	7.15E−08	3.17E−08	1.20E−02	**2.12E−02**
	Oligo	Oligo	2.44E−08	3.25E−08	2.26E−01	2.89E−01
	Astrocyte	Astrocyte	4.62E−08	2.95E−08	5.86E−02	8.79E−02
	Non-neural	Non-neural	1.88E−08	3.55E−08	2.98E−01	3.65E−01
ADHD	Excitatory	L6b FEZF2	1.86E−09	4.30E−09	3.32E−01	3.91E−01
	Excitatory	L5/6 NP FEZF2	2.39E−09	4.48E−09	2.97E−01	3.65E−01
	Excitatory	IT LINC00507 THEMIS RORB	5.76E−09	4.41E−09	9.56E−02	1.33E−01
	Excitatory	L6 CT FEZF2	4.44E−09	4.26E−09	1.49E−01	1.99E−01
	Excitatory	L5/6 IT Car3 THEMIS	3.73E−09	4.30E−09	1.93E−01	2.52E−01
	Excitatory	L5 ET FEZF2	7.33E−10	4.29E−09	4.32E−01	4.98E−01
	Excitatory	L4 IT RORB	8.40E−09	4.35E−09	2.66E−02	**4.43E−02**
	Inhibitory	PAX6	1.13E−10	4.31E−09	4.90E−01	5.43E−01
	Inhibitory	LAMP5	−1.16E−09	4.38E−09	6.04E−01	6.36E−01
	Inhibitory	VIP	3.98E−10	4.50E−09	4.65E−01	5.26E−01
	Inhibitory	SST	−4.92E−10	4.34E−09	5.45E−01	5.84E−01
	Inhibitory	PVALB	2.17E−11	4.51E−09	4.98E−01	5.43E−01
	Oligo	Oligo	−1.70E−09	4.32E−09	6.53E−01	6.76E−01
	Astrocyte	Astrocyte	−2.20E−09	4.50E−09	6.87E−01	6.99E−01
	Non-neural	Non-neural	−4.87E−09	4.36E−09	8.68E−01	8.68E−01
Education	Excitatory	L6b FEZF2	5.16E−09	9.21E−10	1.04E−08	**5.67E−08**
	Excitatory	L5/6 NP FEZF2	4.49E−09	9.68E−10	1.73E−06	**4.51E−06**
	Excitatory	IT LINC00507 THEMIS RORB	5.86E−09	9.71E−10	7.81E−10	**9.37E−09**
	Excitatory	L6 CT FEZF2	5.90E−09	9.27E−10	9.95E−11	**1.49E−09**
	Excitatory	L5/6 IT Car3 THEMIS	5.38E−09	9.42E−10	5.69E−09	**3.79E−08**
	Excitatory	L5 ET FEZF2	5.20E−09	9.74E−10	4.61E−08	**1.98E−07**
	Excitatory	L4 IT RORB	5.81E−09	8.93E−10	3.82E−11	**1.15E−09**
	Inhibitory	PAX6	5.16E−09	9.14E−10	8.21E−09	**4.93E−08**
	Inhibitory	LAMP5	5.75E−09	8.97E−10	7.37E−11	**1.47E−09**
	Inhibitory	VIP	5.93E−09	9.07E−10	2.99E−11	**1.15E−09**
	Inhibitory	SST	4.81E−09	9.12E−10	6.47E−08	**2.43E−07**
	Inhibitory	PVALB	4.85E−09	9.06E−10	4.38E−08	**1.98E−07**
	Oligo	Oligo	3.64E−09	1.00E−09	1.45E−04	**3.35E−04**
	Astrocyte	Astrocyte	3.01E−09	1.07E−09	2.50E−03	**5.17E−03**
	Non-neural	Non-neural	1.40E−09	1.01E−09	8.29E−02	1.21E−01
Cognitive	Excitatory	L6b FEZF2	1.07E−08	2.19E−09	5.31E−07	**1.59E−06**
	Excitatory	L5/6 NP FEZF2	9.95E−09	2.09E−09	9.85E−07	**2.69E−06**
	Excitatory	IT LINC00507 THEMIS RORB	1.10E−08	2.10E−09	7.66E−08	**2.70E−07**
	Excitatory	L6 CT FEZF2	1.12E−08	2.10E−09	5.26E−08	**2.10E−07**
	Excitatory	L5/6 IT Car3 THEMIS	1.11E−08	2.30E−09	7.19E−07	**2.05E−06**
	Excitatory	L5 ET FEZF2	9.23E−09	2.13E−09	7.37E−06	**1.84E−05**
	Excitatory	L4 IT RORB	1.17E−08	2.10E−09	1.28E−08	**6.40E−08**
	Inhibitory	PAX6	1.28E−08	2.16E−09	1.70E−09	**1.50E−08**
	Inhibitory	LAMP5	1.23E−08	2.14E−09	4.56E−09	**3.42E−08**
	Inhibitory	VIP	1.32E−08	2.24E−09	1.75E−09	**1.50E−08**
	Inhibitory	SST	1.04E−08	2.05E−09	2.12E−07	**7.07E−07**
	Inhibitory	PVALB	1.01E−08	2.04E−09	3.46E−07	**1.09E−06**
	Oligo	Oligo	8.87E−09	2.24E−09	3.73E−05	**8.95E−05**
	Astrocyte	Astrocyte	6.46E−09	2.25E−09	2.03E−03	**4.51E−03**
	Non-neural	Non-neural	1.13E−09	2.28E−09	3.09E−01	3.71E−01

Bold indicates significant at FDR .05.

**Table 3:** Cell type enrichment in Kreigstein fetal dataset.

GWAS	Subclass	Coefficient	Std Error	*P*-value	FDR
Word Reading	MGE-derived Ctx inh, Cortical Plate & Germinal Zone enriched	−1.45E−08	3.22E−08	6.74E−01	6.88E−01
	Early and Late Born Exc Neuron V1, Exc Neuron V1 - late born	9.67E−09	3.14E−08	3.79E−01	4.86E−01
	Early and Late Born Exc Neuron PFC	3.90E−08	3.10E−08	1.05E−01	1.87E−01
	CGE/LGE-derived inh neurons	3.38E−08	3.01E−08	1.31E−01	2.03E−01
	Early Born Deep Layer/subplate Exc Neuron V1 and PFC	3.68E−08	3.08E−08	1.16E−01	1.92E−01
	MGE newborn neurons	−5.77E−09	3.45E−08	5.67E−01	6.19E−01
	Newborn Exc Neuron-early and late born	4.17E−08	3.09E−08	8.88E−02	1.70E−01
	Intermediate Progenitor Cells EN-like	−1.70E−08	3.28E−08	6.98E−01	6.98E−01
	MGE Progenitors	4.18E−09	3.38E−08	4.51E−01	5.15E−01
	Dividing Radial Glia (G2/M-phase)	6.17E−08	3.39E−08	3.46E−02	7.55E−02
	MGE Radial Glia 1 & 2	3.67E−08	3.49E−08	1.47E−01	2.21E−01
	Dividing Radial Glia (S-phase), oRG, tRG, vRG	3.86E−08	3.40E−08	1.28E−01	2.03E−01
ADHD	MGE-derived Ctx inh, Cortical Plate & Germinal Zone enriched	1.20E−09	4.53E−09	3.95E−01	4.86E−01
	Early and Late Born Exc Neuron V1, Exc Neuron V1 - late born	3.37E−09	4.71E−09	2.38E−01	3.46E−01
	Early and Late Born Exc Neuron PFC	−1.74E−09	4.63E−09	6.47E−01	6.88E−01
	CGE/LGE-derived inh neurons	5.31E−09	4.42E−09	1.15E−01	1.92E−01
	Early Born Deep Layer/subplate Exc Neuron V1 and PFC	2.03E−09	4.17E−09	3.13E−01	4.17E−01
	MGE newborn neurons	1.68E−10	4.72E−09	4.86E−01	5.43E−01
	Newborn Exc Neuron-early and late born	1.11E−09	5.00E−09	4.12E−01	4.94E−01
	Intermediate Progenitor Cells EN-like	8.69E−10	4.65E−09	4.26E−01	4.99E−01
	MGE Progenitors	2.39E−09	4.62E−09	3.02E−01	4.14E−01
	Dividing Radial Glia (G2/M-phase)	1.29E−09	4.85E−09	3.95E−01	4.86E−01
	MGE Radial Glia 1 & 2	3.08E−09	4.76E−09	2.59E−01	3.66E−01
	Dividing Radial Glia (S-phase), oRG, tRG, vRG	−1.93E−09	4.68E−09	6.60E−01	6.88E−01
Education	MGE-derived Ctx inh, Cortical Plate & Germinal Zone enriched	4.78E−09	1.02E−09	1.49E−06	**7.15E−05**
	Early and Late Born Exc Neuron V1, Exc Neuron V1 - late born	5.32E−09	1.22E−09	6.18E−06	**9.89E−05**
	Early and Late Born Exc Neuron PFC	4.94E−09	1.21E−09	2.08E−05	**2.50E−04**
	CGE/LGE-derived inh neurons	3.75E−09	9.98E−10	8.37E−05	**5.02E−04**
	Early Born Deep Layer/subplate Exc Neuron V1 and PFC	2.96E−09	9.18E−10	6.21E−04	**2.71E−03**
	MGE newborn neurons	4.10E−09	1.29E−09	7.23E−04	**2.89E−03**
	Newborn Exc Neuron-early and late born	4.21E−09	1.29E−09	5.62E−04	**2.70E−03**
	Intermediate Progenitor Cells EN-like	2.96E−09	1.24E−09	8.50E−03	**2.27E−02**
	MGE Progenitors	3.27E−09	1.25E−09	4.39E−03	**1.32E−02**
	Dividing Radial Glia (G2/M-phase)	3.33E−09	1.32E−09	5.92E−03	**1.67E−02**
	MGE Radial Glia 1 & 2	2.87E−09	1.31E−09	1.45E−02	**3.66E−02**
	Dividing Radial Glia (S-phase), oRG, tRG, vRG	2.68E−09	1.27E−09	1.77E−02	**4.25E−02**
Cognitive	MGE-derived Ctx inh, Cortical Plate & Germinal Zone enriched	1.00E−08	2.23E−09	3.47E−06	**8.33E−05**
	Early and Late Born Exc Neuron V1, Exc Neuron V1 - late born	1.21E−08	3.12E−09	5.18E−05	**4.14E−04**
	Early and Late Born Exc Neuron PFC	1.24E−08	3.17E−09	4.25E−05	**4.08E−04**
	CGE/LGE-derived inh neurons	7.69E−09	2.15E−09	1.80E−04	**9.60E−04**
	Early Born Deep Layer/subplate Exc Neuron V1 and PFC	8.36E−09	2.21E−09	7.83E−05	**5.02E−04**
	MGE newborn neurons	9.71E−09	3.13E−09	9.76E−04	**3.60E−03**
	Newborn Exc Neuron-early and late born	8.79E−09	3.23E−09	3.21E−03	**1.03E−02**
	Intermediate Progenitor Cells EN-like	8.73E−09	3.08E−09	2.32E−03	**7.95E−03**
	MGE Progenitors	4.68E−09	3.23E−09	7.35E−02	1.47E−01
	Dividing Radial Glia (G2/M-phase)	5.87E−09	2.98E−09	2.44E−02	5.58E−02
	MGE Radial Glia 1 & 2	5.67E−09	3.40E−09	4.75E−02	9.91E−02
	Dividing Radial Glia (S-phase), oRG, tRG, vRG	3.74E−09	2.99E−09	1.05E−01	1.87E−01

Bold indicates significant at FDR .05.

**Table 4:** Cell type enrichment in Shen fetal dataset.

GWAS	Major Class	Coefficient	Std Error	*P*-value	FDR
Word Reading	Excitatory	4.52E−08	3.40E−08	9.17E−02	1.63E−01
	Inhibitory	2.55E−08	3.55E−08	2.37E−01	2.92E−01
	Intermediate Progenitor	3.78E−08	3.53E−08	1.42E−01	2.28E−01
	Radial Glia	2.59E−08	3.62E−08	2.37E−01	2.92E−01
ADHD	Excitatory	3.08E−09	5.57E−09	2.90E−01	3.31E−01
	Inhibitory	1.60E−09	5.01E−09	3.75E−01	3.99E−01
	Intermediate Progenitor	4.85E−09	6.12E−09	2.14E−01	2.92E−01
	Radial Glia	−2.07E−09	5.37E−09	6.50E−01	6.50E−01
Education	Excitatory	6.31E−09	1.35E−09	1.52E−06	**4.88E−06**
	Inhibitory	6.60E−09	1.25E−09	6.19E−08	**3.30E−07**
	Intermediate Progenitor	4.51E−09	1.18E−09	6.90E−05	**1.84E−04**
	Radial Glia	3.32E−09	1.31E−09	5.51E−03	**1.10E−02**
Cognitive	Excitatory	1.82E−08	2.88E−09	1.33E−10	**2.12E−09**
	Inhibitory	1.57E−08	2.92E−09	3.70E−08	**2.96E−07**
	Intermediate Progenitor	1.42E−08	2.86E−09	3.40E−07	**1.36E−06**
	Radial Glia	1.02E−08	3.13E−09	5.79E−04	**1.32E−03**

Bold indicates significant at FDR .05.

### EWCE

*EWCE* employs ANOVA, through the *‘*drop.uninformative.genes’ function [48]. The mean and specific expression of each gene are then calculated for each cell type using the ‘generate.celltype.data’ function. The first level (“level 1”) for the ANOVA was broader and based on the cell type. The second level (“level 2”) was narrower and based on specific neural markers.

For ABB analysis, level 1 consisted of the four major cell types and level 2 consisted of the 19 subclasses (for example “Inhibitory neuron LAMP5”) (Supplementary Table 1). The ANOVA for both analyses (ABB major and subclasses) was conducted on level 2 because we wanted to include more categories (i.e., fewer categories = fewer dropped genes). The oligodendrocyte precursor cell cluster was put with the oligodendrocyte cluster (Supplementary Table 1).

For the Kriegstein analysis, level 1 consisted of the 12 cell classes shown in Fig. 2 of the supplementary material from that paper. Level 2 was classified using neural markers. The ANOVA was conducted on level 1 because the main analysis was on level 1.

**Fig. 2:**
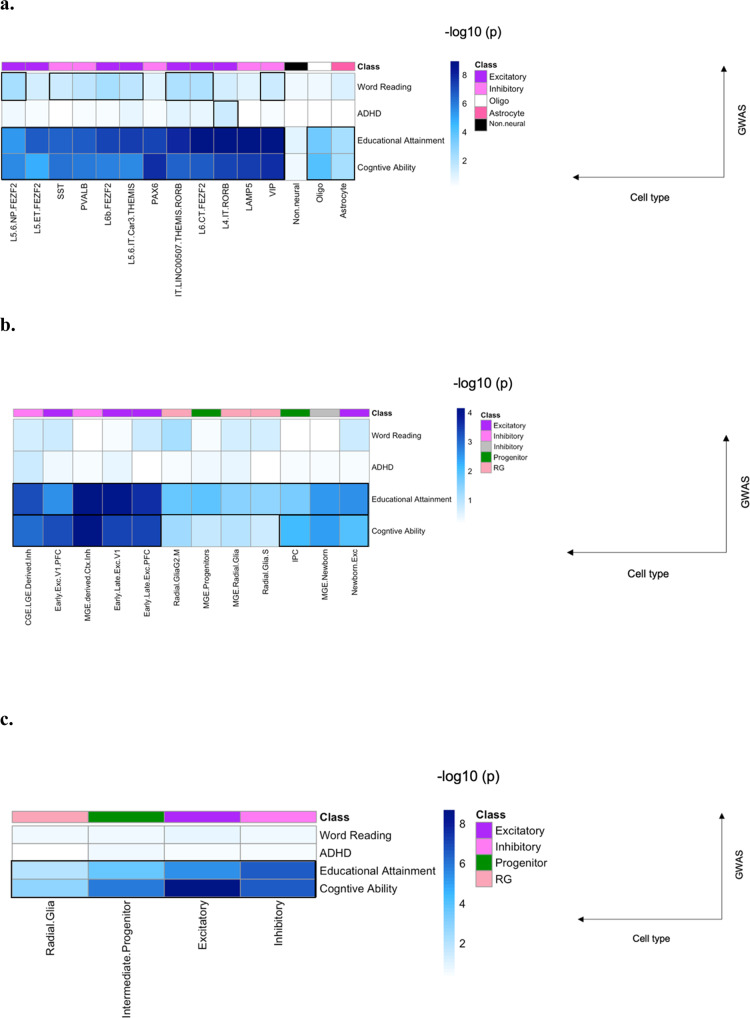
Results of LDSC for Allen Brain Bank, Kriegstein, and Shen RNA sequencing datasets. X-axis are the cell types denoted by layer and gene marker. Y-axis is the GWAS summary statistics used. Class is colour coded, purple are excitatory neurons, pink are inhibitory neurons, white are oligodendrocytes, hot pink are astrocytes, grey are newborn inhibitory neurons, forest green are intermediate progenitor cells, light pink are radial glia (RG), and black are non-neural cells (endothelial, pericytes). The blue is the negative log10 of the FDR q-value (mean analysis). Black boxes represent cell types that reached significance (FDR < 0.05). **a** Allen Brain Bank adult single nuclei RNA sequencing data. **b** Kriegstein fetal single cell RNA sequencing data. **c** Shen fetal RNA sequencing data.

### Gene coordinates

For each annotation file, a gene’s chromosome and transcription start/end position (hg19) +/- 100 kb [45, 48–50] were needed. R packages *biomaRt* and *dplyr* were used to extract this information from ‘ENSEMBL_MART_ENSEMBL’ for each gene ID (ENSEMBL or HGNC Symbol, homo-sapiens). The annotation files were then formatted using the LDSC command ‘make_annot.py’ and phase 3 of the 1000 Genomes Project [70]. The phase 3 of the 1000 Genomes project was used to compute LD scores, and thus it was used for the annotation file to ensure the SNPs matched.

### Partitioning heritability

Partitioning heritability has been described thoroughly by others [45, 46, 48], with the goal being to partition SNP heritability for each cell type. In other words, each cell type annotation file becomes a category for which SNP heritability is calculated and compared to overall SNP heritability. This process permits a test of whether the particular category is enriched within the GWAS results and therefore potentially contributing to its etiology.

LD scores were computed using the command ‘ldsc.py’ and a 1 centiMorgan window, as recommended. This command used only SNPs from the Hapmap3 “print_snps.txt” file corresponding to the baseline annotations – a choice based on the use of the baseline annotations as controls in the cell type analysis, and the need to ensure the matching of the SNP lists.

LD score regressions were run with the aforementioned LD scores. The ‘ldsc.py’ command was used with LD weights calculated from HapMap3 SNPs (“weights_hm2_no_hla”). Weights were used to minimize the standard error by taking into consideration heteroscedasticity and over-counting due to LD [46]. The baseline annotations were used as controls. The “overlap-annot” command was used to account for overlapping regions in the baseline file and the annotation file. The “frqfile-chr” command was used for MAF ( < 5%). LDSC outputs the proportion of SNPs, proportion of heritability, enrichment, standard errors, and regression coefficients. Note that enrichment is proportion of heritability / proportion of SNPs. For computational efficiency, we outputted only the regression coefficients, in order to prioritize cell types [45].

### Threshold for significance

Each RNA seq dataset was corrected separately using a False Discovery Rate (FDR) correction (p < 0.05). The mean and specificity analyses were treated separately.

## Results

### Heritability of GWAS datasets

For the word reading sample, *h*^2^ was approximately 0.25. The *h*^2^ for the other reading-correlated traits, ADHD, educational attainment, and cognitive function have previously been published [59–61]. We recomputed *h*^2^ for these traits and obtained similar results. For ADHD, *h2* was approximately 0.24, educational attainment was 0.11, and cognitive function was 0.18. The z-scores were as follows; word reading 3, ADHD 12, educational attainment 41, and cognitive function 24.

### Analyses of adult neural cells

We began by examining the relationship between word reading/reading-related traits and major brain cell types (excitatory, inhibitory, astrocytes, and oligodendrocytes) in the adult cortex using the ABB dataset and mean gene expression. For word reading, we identified significant enrichment for excitatory and inhibitory neurons (Table 1, FDR < 0.05). For educational attainment and cognitive ability, we identified significant enrichment for excitatory and inhibitory neurons, astrocytes, and oligodendrocyte (Table 1, FDR < 0.05). For ADHD, no significant results were found (Table 1 and Supplementary Table 3). When we next examined specific gene expression, we found enrichment for excitatory neurons for word reading, educational attainment, and cognitive ability. Inhibitory neurons also passed the FDR threshold for significance for cognitive ability (Supplementary Table 3). Non-neural cells were not significant for any reading or related trait for mean or specificity.

We then looked at word reading/reading-related traits and adult cortical cells using the ABB dataset and mean gene expression, but this time using subclasses, defined by their cluster label (marker genes and generally defined by laminar distribution) [63]. For word reading, we identified significant enrichment in select excitatory (L6b FEZF2, L5/6 NP FEZF2, IT LINC00507 THEMIS RORB, L6 CT FEZF2, and L5/6 IT Car3 THEMIS) and inhibitory neurons (VIP, SST, and PVALB) (Table 2, Fig. 2a, FDR < 0.05). For educational attainment and cognitive ability, we identified significant enrichment for all cell types under the categories of excitatory (L6b FEZF2, L5/6 NP FEZF2, IT LINC00507 THEMIS RORB, L6 CT FEZF2, L5/6 IT Car3 THEMIS, L5 ET FEZF2, and L4 IT RORB) and inhibitory (PAX6, LAMP5, VIP, SST, PVALB) neurons as well as astrocytes and oligodendrocytes (Table 2, Fig. 2a, FDR < 0.05). For ADHD, one subclass (L4 IT RORB) was found (Table 2, Fig. 2a, FDR < 0.05). For specific gene expression, only excitatory neurons (IT LINC00507 THEMIS RORB) for educational attainment passed the FDR threshold (Supplementary Table 4). Non-neural cells were not significant for any reading or related trait for mean or specificity.

### Analyses of fetal neural cells

We examined the relationship between word reading/reading-related traits and brain cell types in the fetal cortex using the Kriegstein dataset and mean gene expression. Multiple significant cell types were identified for educational attainment and cognitive ability (Table 3, Fig. 2b, FDR < 0.05). The most significantly enriched cell type was inhibitory neurons from the MGE region of the cortical plate for educational attainment, which was also significant for cognitive ability (Table 3, Fig. 2b, FDR < 0.05). All other cell types were significant for educational attainment. Excitatory and inhibitory neurons and intermediate progenitor cells also reached significance for cognitive ability, but not the radial glial cells or MGE progenitors (Table 3, Fig. 2b, FDR < 0.05). For word reading and ADHD, no significant enrichment was found. Similar results were found across the GWAS for specificity for word reading, cognitive ability, and ADHD (Supplementary Table 5). For specificity and educational attainment, CGE/LGE-derived inhibitory neurons, intermediate progenitor cells, MGE progenitors, and radial glial cells did not reach significance (Supplementary Table 5).

We repeated the examination of word reading/reading-related traits and brain cell types in the fetal cortex using the Shen dataset and mean gene expression. For educational attainment and cognitive ability, results were significant for intermediate progenitor cells, excitatory and inhibitory neurons, and radial glia (Table 4, Fig. 2c). For specific gene expression, the results that passed the threshold were limited to excitatory neurons for educational attainment and cognitive ability (Supplementary Table 6).

## Discussion

Genetic studies are beginning to identify risk variants for reading ability/disability and genetically correlated traits. However, the majority of the heritable variance remains in results that do not reach the threshold for significance. Key questions also remain as to the mechanisms of actions and the neural cell types impacted by risk genes and integral to the reading process. To contribute to understanding the relevant cell types, we used LDSC, a powerful tool, to identify enrichment in cell types for GWAS results for word reading and genetically correlated traits (ADHD, educational attainment, and cognitive ability).

Multiple regions of the cortex have been implicated in the reading process, therefore we chose to use datasets that sampled from these in both adult (anterior cingulate, primary visual, primary motor, primary somatosensory, and primary auditory cortices [63]) and fetal brain (V1, PFC, and MGE [64], and general cortex [65]). The ABB dataset included 722/15206 nuclei from the middle temporal gyrus MTG (L5) region, which is critical for sight word reading [71]. In addition to cell types from different regions of the cortex, we used different types of RNA sequencing datasets, including scRNA-seq, snRNA-seq, and bulk RNA-seq from flow sorted neural cells. Although generally similar in gene expression, differences in cell representation and gene expression between scRNA-seq versus snRNA-seq have been documented [48, 72, 73]. The number of different transcripts identified in whole cells is higher than in nuclei, however some cell types are more vulnerable to the disassociation process in brain tissues (non-neuronal cells survive better than neuronal) and consequently underrepresented in whole cell data [72]. Longer genes are more abundant in nuclear RNA and snRNA-seq is also less susceptible to perturbed gene expression changes that occur during isolation (e.g., immediate early genes) than whole cells [72, 73]. However, genes related to cellular respiration are underrepresented in the nucleus [73].

### Word reading

For the analyses of cell types and word reading, we identified significant enrichment for major classes and subclasses of adult excitatory and inhibitory neurons in the ABB dataset (snRNA-seq). We identified enrichment in three excitatory subclasses with the marker gene *FEZF2* (L6b, L5/L6 NP, L6 CT) and one intratelencephalic (IT) subclass that includes 25 cluster labels (Supplementary Table 1) marked by the genes *LINC00507*, *THEMIS* and *RORB* in layers L3 to L6 (Table 2). We identified three inhibitory subclasses with the marker genes VIP, SST and PVALB.

For word reading, we did not identify significant enrichment for any cell types in the Kriegstein fetal brain datasets (scRNA-seq) or the Shen fetal (bulk seq) data from sorted cells. The number of cells in the Kreigstein dataset was ~4,000 cells compared to ~49,000 nuclei in the ABB adult dataset (snRNA-seq), thus this may be a function of power or the different methods used for the creation of the expression datasets and it is premature to rule out fetal neural cells. It also may be a function of the low *h*^*2*^ for word reading.

To our knowledge, only one cell type has previously been significantly associated with word reading, after correction for multiple testing (correction for 142 cell types) [8]. A multivariate analysis of reading traits (n = 34,000) and RNA-seq data from embryonic, fetal, and adult brain tissues, identified significant enrichment in neurons from the fetal red nucleus – a subcortical structure in the ventral midbrain, part of the olivocerebellar and cerebello-thalamo-cortical systems [8]. The authors suggested this may be due to the fact the red nucleus neurons are more mature, and thus inducing a correlation, compared to the other more fetal neurons in the dataset. The larger study of self-report dyslexia did not identify significant relationships when partitioning by three neural cell types, neurons, astrocytes, or oligodendrocytes [3]. The cell type datasets were from mouse forebrain [74] which may have been a factor, however previous studies have shown high correlations in gene expression for cell type across species (median correlation 0.68) [49] and key cell types found in mouse were replicated in human datasets for brain disorders using LDSC analyses [48, 49].

### ADHD

For the analyses of cell types and ADHD, we identified significant enrichment for one adult excitatory neuron subclass in the ABB dataset (L4 IT RORB). This result was found only when using mean gene expression, whereas no significant enrichment was found for specificity. A previous study that examined this ADHD GWAS data [59] and adult neural cells using gene specificity also did not find significant enrichment for any cell type [49].

### Educational attainment and cognitive ability

Our results show enrichment for multiple cell types in adult brain for educational attainment and cognitive ability. Both traits showed enrichment for astrocytes and oligodendrocytes and all the subclasses of adult excitatory and inhibitory neurons analyzed in the ABB dataset.

Enrichment was also significant in multiple fetal cell types providing new important information for these phenotypes. In the Kriegstein dataset, significant enrichment was identified for educational attainment for all neural subclasses. For cognitive ability, the results for all neurons and intermediate progenitor cells reached significance, but no subclasses for radial glial cells or MGE progenitors (Table 3).

In the Shen fetal dataset, we identified enrichment for fetal excitatory and inhibitory neurons for educational attainment and cognitive ability (Table 4). We also identified significant enrichment in intermediate progenitors and radial glial cells for both phenotypes. Intermediate neural progenitors are migrating neural precursor cells that migrate from the ventricular zone to populate the subventricular zone. They are responsible for the increased neuronal output and expansion and gyrification of the human cortex [75] and thus, relevant for human higher-order cognitive functions.

Skene et al. (2018), Bryois et al. (2020), and Olislagers et al. (2022) have all previously examined educational attainment, cognitive ability, and/or ADHD [48–50]. We add to this literature by examining a word reading GWAS and new fetal RNA sequencing datasets. We also examined multiple subclasses in the adult ABB dataset. Previously, the aforementioned studies used human adult datasets (Habib et al. 2017[76], and Lake et al., 2017/2018 [73, 77]), LDSC, and specific gene expression. Their analyses identified multiple cell types including adult excitatory and inhibitory neurons for educational attainment and intelligence. Our study supports the results of the previous studies. We found adult excitatory and inhibitory neurons for educational attainment and intelligence as well as multiple subclasses using the ABB dataset. We also identified one new adult cell type for ADHD.

Olislagers et al. (2022) is the only study that examined human fetal RNA sequencing data and reached statistical significance using MAGMA v1.08 within the FUMA program. For educational attainment and intelligence, human fetal midbrain and prefrontal cortex GABAergic neurons were enriched (Olislagers Table S14). Fetal prefrontal cortex glutamatergic neurons and fetal quiescent neural stem cells were also enriched. Our study supports these results, as we found fetal excitatory and inhibitory neurons for educational attainment and intelligence. Our study contributes to the literature as the Kriegstein dataset included new subclasses of neural cells which have not previously been examined. We are also the first study to report LDSC using the Shen dataset.

The results of our study identified important cell types for word reading and ADHD and provide new information on fetal cell types for educational attainment and cognitive ability. The findings for word reading and ADHD implicate specific subclasses of excitatory and inhibitory neurons supporting previous data indicating excitatory/inhibitory imbalance in both disorders as indicated by increased cortical glutamate, with higher concentrations of cortical glutamate correlated with lower reading skills [32–34] and ADHD [39–41]. These data further indicate excitatory and inhibitory neurons for both disorders as relevant cell types for study using stem cell derived neurons.

The differences in terms of the number of significant cell types between word reading and ADHD compared to educational attainment and cognitive ability is likely a function of power as these GWAS datasets are substantially smaller (*n* = 5054 for word reading and ~50000 for ADHD) than those for educational attainment (*n* = 1,100,000, minus 23andme sample 776345) and cognitive ability (*n* = 279,930). The available GWAS sample sizes are currently a limitation of the identification of cell types contributing to these disorders. A further limitation is the number of cells/nuclei sequenced for some of the datasets that resulted in some of the cell types dropped from the analyses due to low coverage as well as reduced power to detect gene expression differences. We focused analyses on cortical samples, given the importance of these brain regions in reading. The involvement of cell types in other brain regions were not investigated in our study and are currently unknown. Studies of fetal cells are also limited by the use of combined analyses of samples from different developmental periods which may obscure findings for developmental stage-specific cell types. Caution should be taken when interpreting the results of the word reading meta-analysis given the current sample size. We fully expect additional cell types to be identified with larger GWAS datasets and expanded coverage of cells/nuclei represented in brain.

## Supplementary information

Supplementary Table 1. ABB Cell Type Clusters After Quality Control

Supplementary Table S2. Kreigstein Cell Type Subclasses After Quality Control

Supplementary Table 3. Specificity Analyses ABB Classes

Supplementary Table S4. Specificity Analyses ABB Subclasses

Supplementary Table S5. Kriegstein Specificity

Supplementary Table S6. Specificity Shen Dataset

## Data Availability

Summary statistics for Toronto sample are available upon request. The ADHD, educational attainment, and cognitive ability GWAS are publicly available (see Methods).
